# Combination of Drugs in the Treatment of Alcohol Use Disorder: A Meta-Analysis and Meta-Regression Study

**DOI:** 10.3390/brainsci15060542

**Published:** 2025-05-22

**Authors:** João Vitor Guimarães Mandaji, Maria Olivia Pozzolo Pedro, Kae Leopoldo, João Pini Alemar, Julio Torales, Antonio Ventriglio, João Mauricio Castaldelli-Maia

**Affiliations:** 1 Department of Neuroscience, Medical School, FMABC Health University Center, Santo André 09060-870, Brazil; joao.mandaji@aluno.fmabc.net (J.V.G.M.); joaopalemar@gmail.com (J.P.A.); jmcmaia@usp.br (J.M.C.-M.); 2 Department of Psychiatry, Medical School, University of São Paulo, São Paulo 05508-220, Brazil; maria.oliviapozzolo@gmail.com; 3 Department of Experimental Psychology, Institute of Psychology, University of São Paulo, São Paulo 05508-220, Brazil; kae.leopoldo@usp.br; 4 Department of Medical Psychology, Nacional University of Asunción, Asuncion 111421, Paraguay; jtorales@med.una.py; 5 Department of Clinical and Experimental Medicine, University of Foggia, 71122 Foggia, Italy

**Keywords:** alcoholism, alcohol use disorder, combined pharmacotherapy, abstinence rates

## Abstract

**Background:** Alcohol Use Disorder (AUD) is highly prevalent among substance use disorders worldwide and is characterized by a multifactorial pathophysiology. AUD treatment is mostly based on combined pharmacotherapy and multidisciplinary clinical approaches. Nonetheless, meta-analytical studies assessing the efficacy of combination therapy are scarcely available. **Methods:** We searched for randomized clinical trials through PubMed, ClinicalTrials.gov, Cochrane Library, SciELO, Biblioteca Virtual em Saúde, and Google Scholar databases. Original clinical trials published in English and Portuguese were selected. Data collection followed the PRISMA and MOOSE guidelines and was assessed using the Risk of Bias Tool (RoB 2). Heterogeneity was assessed using the Q test. Meta-regression was conducted using Egger’s regression method. Twelve articles were finally included in the analysis, and random-effects models were applied on aggregate trial results. **Results:** The meta-analysis found that combination therapies led to an average 4.045% increase in abstinence rates (95% CI: 0.415% to 7.675%) compared to monotherapies. Meta-regression showed a strong positive association between the use of naltrexone, acamprosate, and sertraline—either alone or in combination—and treatment success in AUD. The meta-regression also highlighted the impact of patients’ variables, such as gender, age, country, and psychiatric comorbidities, on their treatment outcomes. These findings may identify a potential therapeutic pathway promoting alcohol abstinence, further supported by a Number Needed to Treat (NNT) of 25, as an acceptable value for substance use disorder treatments. **Conclusions:** Combined pharmacotherapies are more effective than monotherapy in enhancing abstinence rates in AUD treatment, with naltrexone, acamprosate, and sertraline emerging as key adjunctive agents promoting these outcomes. These findings underscore the complexity of AUD as a multifactorial psychiatric condition and highlight the potential of combined pharmacotherapy as a promising strategy for achieving better treatment outcomes, particularly in terms of abstinence rates.

## 1. Introduction

In 2020, the National Survey on Drug Use and Health reported that more than 28.3 million people aged 12 years or older in the United States (U.S.) met the DSM-5 (Diagnostic and Statistical Manual of Mental Disorders, Fifth Edition) criteria for AUD in the past year [1]. Research evidence confirms that the personal and societal impact of AUD can be mitigated through treatments, which include psychological, social, and pharmaceutical interventions aimed at reducing alcohol consumption and its associated consequences [2]. Despite this evidence, the epidemiological research highlights that 74.5% of individuals with AUD in the U.S. are not receiving or seeking specific treatment, representing a critical gap in health care [3].

AUD is classified as a chronic, relapsing medical condition with a multifactorial etiology including genetic, neurobiological, psychological, and environmental factors [4]. The neurobiological substrate of AUD is characterized by a complex dysregulation across multiple neurotransmitter systems (Gamma-Aminobutyric Acid, Glutamate, Dopamine, Serotonin, Norepinephrine, Endogenous Opioids, Nicotinic Receptors, Endocannabinoid System) mediating the reward effect and abuse liability [5]. Given its complexity, combining medications may enhance treatment outcomes by targeting different neurobiological pathways and addictive behaviors. For instance, combining drugs can simultaneously address positive reinforcement (e.g., the pleasurable effects of alcohol) and negative reinforcement (e.g., relief from withdrawal symptoms), treat comorbid psychiatric and medical conditions, and minimize side effects by using lower doses of each medication [6,7].

The primary goal of AUD treatment should be to support patients in attaining either complete abstinence from alcohol or a substantial reduction in alcohol-related harm through decreased consumption [8]. Although there is an ongoing debate about whether abstinence or harm reduction should be the preferred treatment targets, both approaches have been acknowledged as beneficial [9]. Research suggests that complete abstinence is considered the most favorable outcome; however, even a reduction in alcohol consumption can lead to significant improvements in both general health and quality of life [10,11].

Several pharmaceutical options are available for AUD treatment. For instance, in the U.S., the Food and Drug Administration (FDA) approved the use of disulfiram, naltrexone, and acamprosate, alongside other off-label options, including nalmefene, baclofen, and topiramate, as suggested by successful clinical trials [12]. These trials reported their efficacy in the reduction of craving, prevention of relapses, and mitigation of withdrawal symptoms [12].

While monotherapies, e.g., disulfiram, have shown limited efficacy in preventing relapses beyond short-term abstinence [12,13,14], there is growing interest in combining drugs, such as disulfiram with anti-craving medications like naltrexone and acamprosate, for long-term therapeutic effects [14,15]. Decreasing craving may improve patients’ treatment adherence while also promoting their sustained abstinence [16]. Preclinical studies support the evidence of potential additive effects of drug combinations, as each medication targets distinct neurobiological pathways: naltrexone attenuates the positive reinforcing effects of alcohol, while acamprosate mitigates its negative reinforcing properties [15,16]. Overall, the heterogeneity in methodologies, assessments of AUD clinical severity, outcome measures, and adjunctive psychosocial treatments across studies on combined therapies all represent a significant limitation in the evaluation of their effectiveness [15,16,17].

This meta-analysis and meta-regression study aims to explore the evidence on the impact of combined therapy in the treatment of Alcohol Use Disorder (AUD).

## 2. Materials and Methods

### 2.1. Review Guidelines and Registration

This study followed the PRISMA statement for the transparent report of systematic reviews and meta-analysis [18] and the MOOSE guideline for Meta-analysis of Observational Studies [19]. Each clinical trial was evaluated by the Risk of Bias Tool (RoB 2) (Table 1) [20]. Tables S1 and S2 respectively present PRISMA and MOOSE checklists, indicating the manuscript sections where each item was addressed. This study was registered at the Center for Open Science/Open Science Framework: https://osf.io/fny54/?view_only=1c3c62de4e6f44198aafe6f2343b15db, accessed on 2 May 2025.

### 2.2. Information Sources

We employed the PubMed (U.S. National Library of Medicine), Google Scholar, and ClinicalTrials.gov databases to identify relevant articles on the research topic published in English and Portuguese. The last search considered was performed on 20 December 2024.

The search was based on the following keywords in the title/abstract: “((alcoholism OR alcohol use disorder OR alcohol abuse) combined pharmacotherapy)” filtered by categories “Randomized Controlled Trial” and “Clinical Trial”. Relevant articles were selected through a three-step process. In Step 1, the first author reviewed 991 studies and selected studies focusing on the effects of combined pharmacotherapy on AUD. In Step 2, two authors (the first and last authors) independently evaluated the abstracts selected in Step 1. Finally, in Step 3, articles were included based on consensus between the first and last authors, applying the following criteria: original studies reporting effect size estimates for monotherapy interventions (serving as the control condition) and combined therapy interventions (serving as the experimental condition) in relation to their impact on levels of abstinence; trials reporting data on pharmacological interventions for AUD; trials specifically addressing combined pharmacotherapy; and clinical trials published in English or Portuguese. Also, all studies that did not fulfill the following requirements were excluded: clinical trials conducted on humans, presence of a control group, and inclusion of the clinical parameter ‘percentage of abstinent days’. Additionally, the reference lists of all selected articles were reviewed to identify any missing studies and ensure the completeness of the literature search.

The primary objective of this selective process was to analyze the effect of combined pharmacotherapy for AUD.

Meta-regression analysis was conducted following the systematic tabulation of all included studies. Only variables consistently reported across all studies were considered, ensuring comparability and suitability for inclusion in the regression model.

### 2.3. Data Extraction

Data was extracted from the full-text articles by the first author and reviewed by the last author. All divergencies between the two authors were resolved through discussion with the senior author.

### 2.4. Quality Assessment

The methodological quality of all included studies was assessed using the modified Cochrane Collaboration tool for risk of bias in randomized controlled trials version 2 (RoB 2) [20]. Bias was evaluated across five domains: selection, performance, attrition, reporting, and other potential biases. Among the studies, 11 scored “low risk” and 1 as “some concerns”. All were included in the meta-analysis.

### 2.5. Data Analysis

Data analysis was performed using R software version 3.5.0. The mean difference in the percentage of days abstinent between combined pharmacotherapy and monotherapy was calculated. Heterogeneity was assessed using the Q test and quantified with the I2 statistic, where values below 25% indicated low heterogeneity, 50% indicated moderate heterogeneity, and ≥75% indicated high levels of heterogeneity (Chiang et al.) [21]. The DerSimonian-Laird estimator was used for tau2, and significant heterogeneity was detected.

Subgroup analyses were conducted to explore the influence of variables such as percentage of male participants, mean age, country of trial, psychiatric comorbidities, and drugs used as control or in combination. Publication bias was defined as *p*-values of 0.05 or less, and the ‘trim-and-fill’ technique was applied to adjust for bias, re-estimating the overall effect size and missing studies in the funnel plot (Idris, 2012; Shi and Lin, 2019) [22,23].

Univariate meta-regression analysis was performed to test the association of individual variables with treatment effects, followed by a random-effects regression model used to account for variability across studies (Foo et al., 2018) [24]. The random-effects model enabled generalization of findings by assuming the included trials represent a broader population. A significant level of 5% was applied in all analyses.

**Table 1:** Risk of Bias Tool (RoB2). Naltrexone (NTX); Sertraline (SERT); Topiramate (TPM); Quetiapine (QTP); Disulfiram (DSF); Escitalopram (ESC); Acamprosate (ACAM); Citalopram (CIT); Low Risk (LR); Some Concerns (SC); High Risk (HR); D1 = Randomization process; D2 = Deviations from the intended interventions; D3 = Missing outcome data; D4 = Measurement of the outcome; D5 = Selection of the reported result.

Unique ID	Study ID	Experimental	D1	D2	D3	D4	D5	Overall
1	Helen M Pettinati, 2010 [25]	NTX + SERT	LR	LR	LR	LR	LR	LR
2	Melissa DelBello, 2010 [26]	QTP + TPM	LR	LR	HR	LR	LR	SC
3	Ismene Petrakis, 2007 [27]	NTX + DSF	LR	LR	LR	LR	LR	LR
4	Ismene Petrakis, 2005 [28]	NTX + DSF	LR	LR	LR	LR	LR	LR
5	Ismene Petrakis, 2006 [29]	NTX + DSF	LR	LR	LR	LR	LR	LR
6	Conor K Farren 2008 [30]	NTX + SERT	LR	LR	LR	LR	LR	LR
7	Janet Witte 2013 [31]	ESC + ACAM	LR	LR	LR	LR	LR	LR
8	O’Malley SS, 2008 [32]	NTX + SERT	LR	LR	LR	LR	LR	LR
9	Raymond F Anton, 2006 [33]	NTX + ACAM	LR	LR	LR	LR	LR	LR
10	Adamson SJ, 2015 [34]	NTX + CIT	LR	LR	LR	LR	LR	LR
11	Josep Guardia, 2011 [35]	NTX + QTP	LR	LR	LR	LR	LR	LR
12	Jacques Besson, 1998 [36]	ACAM + DSF	LR	LR	LR	LR	LR	LR

## 3. Results

Twelve studies were finally included in this review, as illustrated in Table S1 (PRISMA flowchart). From an initial pool of 6540 studies, 45 duplicates were excluded, and 991 titles and abstracts were screened. Following the title and abstract review, 72 studies underwent full-text analysis, of which 60 were excluded for lack of clinical parameters of interest, absence of control groups, lack of recorded results, or trials conducted on animals rather than humans. Table 2 summarizes the population characteristics of each trial, while Table 3 presents the main results, organized into comparisons between combined therapy and monotherapy, subgroups with and without psychiatric comorbidities, and groups receiving combined pharmacotherapy with or without psychotherapy.

The risk of bias assessment using the RoB 2 tool (Table 1) indicated that eleven of the twelve included studies presented a low risk of bias across all domains, suggesting a high level of methodological quality and reinforcing the internal validity of the meta-analysis. This consistency supports the reliability of the pooled effect estimates, as the influence of systematic errors related to study design or execution is likely minimal. One study, however, showed a high risk of bias in a single domain while maintaining a low risk in the others. Although this may introduce some concern, its isolated nature and limited extent reduce the likelihood of substantial impact on the overall findings. Nevertheless, the presence of any high-risk study warrants caution, particularly if it contributes significantly to the overall effect size. In this context, complementary analyses such as sensitivity or subgroup analyses based on the risk of bias are recommended to ensure the robustness of the results and to clarify the influence of methodological variability on the conclusions.

Most trials (7 out of 12) were conducted in North America. Diagnostic criteria for AUD were predominantly based on DSM-IV [37] (11 studies) and DSM-III [38] (1 study). Studies included were published between 1998 and 2015, with one from the 1990s, six from the early 2000s, and five from the 2010s. The meta-analysis included 3203 participants, of whom 875 reported psychiatric disorders (e.g., depression, bipolar mania, post-traumatic stress disorder) and 2429 received adjunctive psychological therapy.

**Figure 1:**
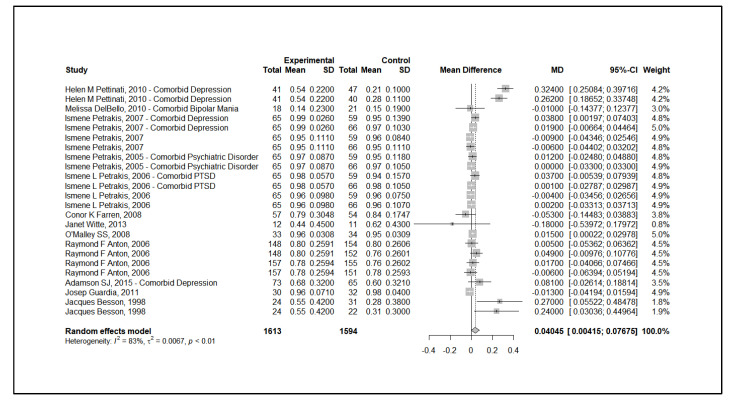
Forest Plot of the Effect Size of Combined Pharmacotherapy versus Monotherapy. Forest plot displaying the mean difference in percentage of abstinent days across studies comparing combined pharmacotherapy and monotherapy. The overall effect favors combined therapy, with a mean difference of 4.045% (95% CI: 0.415% to 7.675%). High heterogeneity was observed (I^2^ = 99.68%) [25,26,27,28,29,30,31,32,33,34,35,36].

The meta-analysis revealed a mean difference of 4.045% (95% CI: 0.415% to 7.675%) in percentage of days abstinent, in favor of combined therapy over monotherapy, as shown in the forest plot (Figure 1). However, significant heterogeneity was detected (I^2^ = 99.68%), and subgroup analysis indicated variability in treatment effects based on participant characteristics, location of trial, and study design.

Of 24 comparisons made, 12 involved naltrexone combined with other drugs (e.g., disulfiram, acamprosate, sertraline, quetiapine, or citalopram) versus naltrexone monotherapy. There were 8 comparisons that reported negative mean differences, indicating better outcomes for monotherapy. Only 6 of the 15 comparisons with positive mean differences reported standard deviations entirely within the positive axis, favoring combined therapy.

**Figure 2:**
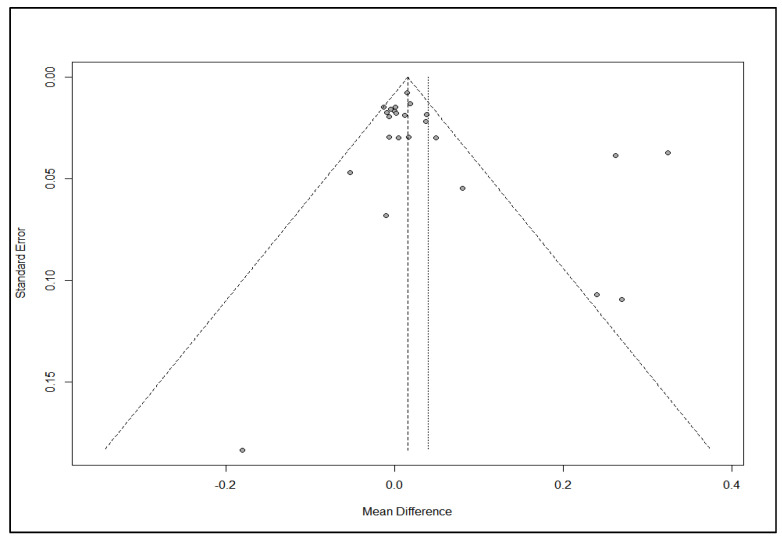
Funnel plot for publication bias assessment. Funnel plot evaluating publication bias across included studies measuring the effect of combined pharmacotherapy compared to monotherapy on alcohol abstinence rates. The Egger’s test regression line is plotted, with the *p*-value suggesting no significant asymmetry (*p* = 0.0823), indicating absence of strong publication bias. Model: weighted regression with multiplicative dispersion. Predictor: standard error Egger’s regression test of funnel plot asymmetry (t = 1.8208, df = 22, *p*-value = 0.0823). Limit estimate (as sei → 0): b = −0.0111 (CI: −0.0471, 0.0250).

As depicted in Figure 2, the funnel plot showed a relatively symmetrical distribution of studies around the regression line, with no strong evidence of publication bias according to Egger’s test (*p* = 0.0823).

Results from the meta-regression (Table 4) identified significant positive associations with the combined use of naltrexone, acamprosate, disulfiram, sertraline, and topiramate, which enhanced patients’ abstinence rates. Conversely, the covariates “percentage male population” and “regions (U.S. vs. other)”—representing the meta-regression of the challenge “trial performed in the U.S.” versus “trial performed in any other country”—reported significant negative associations, suggesting higher effectiveness of combined therapy in populations with a lower proportion of males or in non-U.S. trials. Psychiatric comorbidities and mean age of participants also showed positive associations with improved treatment outcomes, indicating that the mean age of the trial population and the presence of comorbidities in the group of baseline characteristics significantly affect the impact of combined therapy over alcoholism treatment.

The overall analysis indicates that drug combinations yield better outcomes for AUD treatment, measured as percentage of days abstinent. Table 5 reports the Mixed-Effects Model and heterogeneity test (I^2^ = 99.68%) and indicates a substantial degree of heterogeneity, suggesting that the variability in effect sizes across studies is largely attributable to real differences in study characteristics—such as populations, interventions, or methodologies—rather than to random error. While I^2^ is informative for quantifying heterogeneity, its interpretability is limited, as it does not elucidate the source of variation. Therefore, the high heterogeneity observed should be interpreted cautiously and complemented by additional measures, such as τ^2^, alongside an assessment of its clinical relevance. Both combined therapies and monotherapy showed significant associations in improving abstinence rates, reflecting variability in treatment effects.

Groups’ characteristics were identified as significant covariates influencing outcomes in favor of abstinence rates, as covariates ‘mean age’ and ‘patients’ psychiatric comorbidity’, or against as covariates ‘percentage of male patients’ and ‘country in which the trial was conducted (U.S. vs Other)’. Table 4 also presents the residual heterogeneity (τ^2^ = 0.0298), with an explained variability of 89.12% (R^2^), indicating that covariates contributed substantially to differences in effect sizes.

### Key Metrics

- Tau-squared (τ^2^): The residual heterogeneity (τ^2^ = 0.0298) indicates variability in the effect sizes that remains unexplained by the model.
- I^2^ (Residual Heterogeneity/Unaccounted Variability): The I^2^ value is extremely high (99.68%), suggesting that nearly all the variability in the prevalence estimates is due to heterogeneity between studies, not to sampling error.
- H^2^ (Unaccounted Variability/Sampling Variability): H^2^ = 314.52 is quite large, indicating that there is substantial heterogeneity in the mean difference of the effect of combined therapy over monotherapy in relation to the percentage of abstinence parameter across studies. This suggests that there are unmeasured factors causing differences in this parameter, and the model does not fully account for the variability observed.
- R^2^ (Amount of Heterogeneity Accounted for): The R^2^ value is 89.12%, suggesting that the covariates in the model are meaningful in explaining the differences in effect sizes across studies.
- Test for Residual Heterogeneity: The very small *p*-value (<0.0001) indicates there is still significant heterogeneity left unexplained by the covariates, indicating that other factors not included in the model may be contributing to the variability in effect sizes.

## 4. Discussion

We conducted a systematic review and meta-regression of randomized clinical trials in order to evaluate the effect of combined pharmacotherapy compared to monotherapy in AUD treatment. The meta-analysis provided evidence that drug combination results in a modest but significant improvement in patients’ abstinence rates, with a mean difference of 4.04% (95% CI: 0.415% to 7.675%) in the clinical parameter “% days abstinent”.

Although this effect may appear small, it holds clinical relevance. In fact, AUD treatment may be complex due to its multifactorial etiology, encompassing neurobiological, genetic, psychological, social, and environmental factors [17]. Furthermore, the effect corresponds to a Number Needed to Treat (NNT) of 25, which can be considered acceptable in the context of chronic disease treatment, such as AUD, especially if the benefits of the treatment outweigh the risks and costs and if aligning with patients’ preferences [39]. Comparatively, the meta-analysis by McPheeters et al. [40] indicates that the number needed to treat (NNT) to prevent relapse to alcohol use is 11 for acamprosate and 18 for oral naltrexone at a dose of 50 mg/day. Furthermore, to prevent the return to heavy drinking, the NNT for oral naltrexone is 11. On the other hand, the meta-analysis by Jonas et al. [41] reports an NNT of 12 for acamprosate and 20 for oral naltrexone to prevent relapse to alcohol use. To prevent the return to heavy drinking, the NNT for oral naltrexone is 12. It is also important to highlight that when comparing these two meta-analyses with the one provided in this study, there is a key difference: unlike the previous analyses, which derived from the comparison of studies based on therapy versus no therapy, the present analysis provides an NNT for the comparison between combined therapy and monotherapy. Therefore, the value of this parameter should be considered from a different perspective than those obtained in the other two studies. In this context, an NNT of 25 can be regarded as clinically significant, particularly if the treatment is safe, well-tolerated, and considers the substantial impact of Alcohol Use Disorder (AUD) on quality of life and mortality.

From a neurobiological perspective, AUD involves dysregulation in brain regions associated with motivation, stress regulation, and reward, such as the midbrain, limbic system, prefrontal cortex, and amygdala [42,43]. Both positive and negative reinforcement mechanisms play crucial roles in sustaining drinking behavior. Positive reinforcement refers to the rewarding and desirable effects of alcohol, while negative reinforcement involves the relief of negative emotional and physiological states, such as anxiety, depression, or withdrawal symptoms, through alcohol consumption [44].

Combined therapies may target these mechanisms through complementary actions. For example, naltrexone reduces positive reinforcement by blocking opioid receptor-mediated dopamine release in the nucleus accumbens, amygdala, and forebrain [45,46], while also enhancing alcohol sedative effects and reducing cravings triggered by consumption or alcohol-related cues [47].

Acamprosate, a structural analog of the amino acid taurine, reduces alcohol consumption and alleviates withdrawal symptoms by reducing alcohol cravings by modulating NMDA (N-Methyl-D-Aspartate) receptors and inhibiting excitatory glutamatergic activity [48,49]. This action diminishes withdrawal distress and suppresses brain hyper-excitability associated with ethanol consumption [48]. Additionally, experimental studies have shown that acamprosate enhances GABA (Gamma-Aminobutyric Acid) reuptake and modulates dopaminergic activity in the nucleus accumbens, reducing positive reinforcement from alcohol [50,51,52,53].

Disulfiram deters alcohol consumption by inhibiting acetaldehyde dehydrogenase, leading to the accumulation of acetaldehyde in the bloodstream [53,54]. This causes adverse effects, such as flushing, tachycardia, and nausea, which act as strong deterrents to alcohol consumption [55].

Having delineated certain mechanisms of action underlying the pharmacological agents employed in the treatment of Alcohol Use Disorder (AUD), the central tenet of this study rests upon the premise that drug combinations may enhance therapeutic efficacy through two principal strategies. The first strategy posits that both interventions target the same drinking behavior, thereby amplifying their impact. Given that existing treatments exhibit, at best, only moderate effect sizes, this approach emerges as a logical means to bolster clinical outcomes. Ideally, such combinations would yield additive—or even synergistic—effects, though the possibility of adverse interactions cannot be disregarded. This rationale extends not only to pharmacotherapy coupled with behavioral interventions but also to the concomitant administration of two pharmacological agents. For example, a pharmacological agent reducing the positive reinforcement of drinking (e.g., naltrexone) can be paired with a medication that attenuates protracted alcohol withdrawal symptoms (e.g., acamprosate) [17]. These recommendations align with the findings of our study, which indicate strong positive associations between the use of naltrexone, acamprosate, and sertraline, both when combined with other drugs and when other drugs are added to them in the treatment of AUD [Naltrexone associated (estimate: 2.6221, *p* ≤ 0.0001), Acamprosate associated (3.0294, *p* ≤ 0.0001), Sertraline associated (4.2262, *p* ≤ 0.0001), Naltrexone control (1.8796, *p* ≤ 0.0001), Acamprosate control (2.2960, *p* = 0.0002), Sertraline control (3.2263, *p* ≤ 0.0001)].

It is of note that the combination of medications may also increase the risk of adverse drug interactions. For instance, disulfiram, when administered with other medications, can exacerbate pharmacological interactions, particularly with central nervous system stimulants [56]. Furthermore, some studies have reported the occurrence of adverse cardiac events with the combination of naltrexone and disulfiram [57].

Despite the findings of our analysis, there is no consolidated evidence of significant synergistic or additive effects of certain combined medications. For example, pre-clinical studies have not demonstrated additive effects when semaglutide is combined with varenicline or bupropion [58]. This suggests that, in some cases, the combination of medications does not provide additional benefits compared to monotherapy.

Also, polypharmacy may potentially reduce patients’ adherence to treatment; adherence is a critical factor for the successful treatment of substance use disorders [59]. Thus, combined medication regimens may lead to treatment discontinuation.

Finally, treatment combination safety is not thoroughly investigated, especially in populations with comorbidities [57].

In this context, our meta-regression revealed that the variable “% Male” showed a strong negative association (−8.6545, *p* = 0.0095) with the percentage of men in the sample, suggesting that groups with a higher proportion of women tend to show better treatment outcomes. The impact of gender on the treatment outcome of AUD is controversial, with evidence indicating that women more frequently seek help and engage in self-care while tending to drop out of treatment [60]. Also, women are more likely to be responsive and committed to the treatment process [61].

Location of trials showed a significantly negative association (−3.9626, *p* < 0.0001) when comparing studies conducted in the U.S. to those conducted in the other countries (UK, Spain, Switzerland). These differences may reflect the interplay between culturally specific interpretations of mental disorders and the standardized classificatory framework used in the countries (DSM-III and DSM-IV). Current definitions of alcohol dependence and alcohol use disorders are based on culturally specific criteria [62]. Also, the clinical dimensions and subjective experiences of “Alcohol Use Disorders” are often objectives of measurement as well as interpretation [63]. Considering cross-cultural differences may also help in understanding the relationship between alcohol and harm—whether physical, mental, or social—and may suggest different pathways for harm prevention. This perspective helps to partially explain the high heterogeneity observed in the meta-regression (I^2^ = 99.68%) and the associations with the country where the trial was conducted. It may suggest that there may be greater social support for alcohol use treatment in synergy with pharmacological therapy in “non-USA” countries.

We also found a weak but relevant positive association between the rate of psychiatric comorbidities and treatment outcomes (0.2766, *p* = 0.0112). The association between the employment of combination treatments for AUD and comorbidities is still controversial. Pettinati et al. [25] have shown that sertraline and naltrexone demonstrated higher efficacy in individuals with Alcohol Use Disorder (AUD) and concomitant depression. In fact, when compared to sertraline, naltrexone, or placebo administered alone, this combined regimen resulted in a significantly higher incidence of alcohol abstinence and an extended duration until relapse occurred.

Moreover, in those patients who were daily smokers and heavy alcohol consumers, the combination of varenicline and naltrexone was effective in reducing the levels of alcohol consumption. This pharmacological combination reduced the risk of alcohol use compared to varenicline administered alone [64]. Castillo-Carniglia et al. [65] argued that the comorbidity between AUD and psychiatric conditions may be controversial, with AUD leading to additional psychopathological issues as well as mental distress leading to AUD. This still confirms that integrated treatments for alcohol use disorder and comorbid psychiatric disorders lead to better outcomes than non-integrated treatments [66,67,68]. These conclusions align with the results of our meta-regression, reporting a significant correlation between combined treatments and psychiatric comorbidities. Nonetheless, evidence in the literature, including our findings, may be limited since the studies available are based on small sample sizes, short follow-up periods, non-experimental designs, and highly heterogeneous and poorly described treatments [65].

Finally, the meta-regression analysis revealed a positive and significant association between the age of study participants and their treatment outcome (0.2519, *p* = 0.0095). Supporting this finding, a secondary evaluation of the COMBINE (Combined Pharmacotherapies and Behavioral Interventions for Alcohol Dependence) study identified age as one of the primary factors, alongside the number of consecutive days of abstinence prior to randomization, in predicting abstinence from heavy alcohol consumption [69,70]. This suggests that age may influence treatment responses for AUD, with older individuals tending to exhibit better outcomes in terms of sustained abstinence from intensive drinking.

The PREDICT (Predictors of Response to Treatment in Alcoholism) study validated the results of the COMBINE study within a large population in Germany, confirming age as a relevant predictive factor. In fact, the deterministic forest model, based on clinical and statistical criteria, highlighted age as a key factor, alongside the family history of alcoholism and confidence in resisting consumption during periods of abstinence and craving. The external validation of these findings in the PREDICT study further underscores the significance of age as a predictive factor for treatment outcomes across different populations [70].

Although combined pharmacotherapy demonstrated a modest but clinically significant improvement in abstinence rates, it is important to consider pharmacoeconomic implications. Combination therapies may increase direct treatment costs due to the simultaneous use of multiple medications, potentially impacting healthcare budgets and patient adherence. However, these higher initial costs could be offset by long-term benefits, such as reduced relapse rates, fewer hospitalizations, and decreased burden on healthcare services. Evidence from broader substance use treatment literature suggests that interventions improving abstinence are generally cost-effective when accounting for reductions in morbidity, productivity loss, and societal costs [71]. Future studies should directly evaluate the cost-effectiveness of combined pharmacotherapy strategies for Alcohol Use Disorder, considering both direct medical expenses and indirect societal benefits.

## 5. Conclusions

The treatment of AUD reports higher efficacy when based on combined pharmacotherapy compared to monotherapy, particularly including naltrexone, acamprosate, and sertraline. These findings highlight a promising therapeutic approach that enhances alcohol abstinence, further supported by an NNT of 25, underscoring the potential of combination therapies to significantly improve abstinence rates and yield clinically meaningful benefits. Meta-regression analyses emphasize the necessity of considering individual and population-specific factors—such as gender, age, geographic region of investigation, and patient’s psychiatric comorbidities—when tailoring treatment strategies. Despite these advantages, the observed heterogeneity across studies suggests that unmeasured variables, including psychosocial and cultural influences, may play a pivotal role in treatment outcomes. Future research should focus on addressing these gaps to refine and optimize therapeutic strategies for AUD.

## Figures and Tables

**Table 2:** Characteristics of the study sample. United States of America (USA); United Kingdom (UK); Cognitive Behavioral Therapy (CBT); Relapse Prevention (RP); Post-Traumatic Stress Disorder (PTSD); Diagnostic and Statistical Manual of Mental Disorders (DSM); Naltrexone (NTX); Sertraline (SERT); Topiramate (TPM); Quetiapine (QTP); Disulfiram (DSF); Escitalopram (ESC); Acamprosate (ACAM); Citalopram (CIT).

Author	Drug Association	Country	% Male	Comorbid	Mean Age (Years)	Treatment Duration	Measur ED	Psycotherapy Suport (Ps)
Helen M Pettinati (2010) [25]	NTX + SERT	USA	62.40%	Major Depression	43.3	14 week	DSM IV	CBT
Melissa DelBello (2010) [26]	TPM + QTP	USA	38.50%	Bipolar Mania	17.9	12 weeks	DSM IV	No Ps
Ismene Petrakis (2007) [27]	NTX + DSF	UK	97.20%	Depression Group and No Depression Group	47	12 weeks	DSM IV	CBT
Ismene Petrakis (2005) [28]	NTX + DSF	UK	97.20%	Psychiatric Disorders	47	12 weeks	DSM IV	CBT
Ismene Petrakis (2006) [29]	NTX + DSF	UK	97.20%	PTSD Group and No PSTD Group	47	12 weeks	DSM IV	CBT
Conor K Farren (2008) [30]	NTX + SERT	USA	81.96%	No Associated Comorbidities	43.15	11 weeks	DSM IV	RP
Janet Witte (2013) [31]	ESC + ACAM	USA	57%	No Associated Comorbidities	46.13	12 weeks	DSM IV	CBT
O’Malley SS (2008) [32]	NTX + SERT	USA	66%	No Associated Comorbidities	41.2	16 weeks	DSM IV	CBT
Raymond F Anton (2006) [33]	NTX + ACAM	USA	69.16%	No Associated Comorbidities	44.2	16 weeks	DSM IV	CBT Group and No CBT Group
Adamson SJ (2015) [34]	NTX + CIT	USA	40.60%	Depression	43.6	12 weeks	DSM IV	CBT
Josep Guardia (2011) [35]	NTX + QTP	Spain	80.60%	No Associated Comorbidities	43.8	12 weeks	DSM IV	No Ps
Jacques Besson (1998) [36]	ACAM + DSF	Switzerland	80%	No Associated Comorbidities	42.4	360 days	DSM III	No Ps

**Table 3:** Main results of the included studies divided for drug combinations and comorbid groups. Naltrexone (NTX); Sertraline (SERT); Topiramate (TPM); Quetiapine (QTP); Disulfiram (DSF); Escitalopram (ESC); Acamprosate (ACAM); Citalopram (CIT); Placebo (PLA).

Author	No. Total	Comorbid	Association Compaired		n Intervention	n Control	% Days Abstinent (Intervention)				% Days Abstinent (Control)			
			Intervention	Control			Baseline	(SD)	Outcome	(SD)	Baseline	(SD)	Outcome	(SD)
Helen M Pettinati (2010) [25]	88	Major Depression	NTX + SERT	NTX + PLA	41	47	-	-	53.7%	22%	-	-	21.3%	10%
Helen M Pettinati (2010) [25]	81	Major Depression	NTX + SERT	SERT + PLA	41	40	-	-	53.7%	22%	-	-	27.5%	11%
Melissa DelBello (2010) [26]	39	Bipolar Mania	TPM + QTP	QTP + PLA	18	21	-	-	14%	23%	-	-	15%	19%
Ismene Petrakis (2007) [27]	139	Major Depression	NTX + DSF	NTX	65	59	-	-	99%	2.6%	-	-	95.2%	13.9%
Ismene Petrakis (2007) [27]	139	Major Depression	NTX + DSF	DSF + PLA	65	66	-	-	99%	2.6%	-	-	97.1%	10.3%
Ismene Petrakis (2007) [27]	115	No Comorbid	NTX + DSF	NTX	65	59	-	-	94.9%	11.1%	-	-	95.8%	8.4%
Ismene Petrakis (2007) [27]	115	No Comorbid	NTX + DSF	DSF + PLA	65	66	-	-	94.9%	11.1%	-	-	95.5%	11.1%
Ismene Petrakis (2005) [28]	124	Psychiatric Disorders	NTX + DSF	NTX	65	59	-	-	96.6%	8.7%	-	-	95.4%	11.8%
Ismene Petrakis (2005) [28]	131	Psychiatric Disorders	NTX + DSF	DSF + PLA	65	66	-	-	96.6%	8.7%	-	-	96.6%	10.5%
Ismene L Petrakis (2006) [29]	93	PTSD	NTX + DSF	NTX	65	59	-	-	97.8%	5.7%	-	-	94.1%	15.7%
Ismene L Petrakis (2006) [29]	93	PTSD	NTX + DSF	DSF + PLA	65	66	-	-	97.8%	5.7%	-	-	97.7%	10.5%
Ismene L Petrakis (2006) [29]	161	No Comorbid	NTX + DSF	NTX	65	59	-	-	96.1%	9.8%	-	-	96.5%	7.5%
Ismene L Petrakis (2006) [29]	161	No Comorbid	NTX + DSF	DSF + PLA	65	66	-	-	96.10%	9.8%	-	-	95.9%	10.7%
Conor K Farren (2008) [30]	111	No Comorbid	NTX + SERT	NTX + PLA	57	54	29.3%	19.13%	79.2%	30.48%	25.6%	18.66%	84.5%	17.47%
Janet Witte (2013) [31]	23	No Comorbid	ESC + ACAM	ESC + PLA	12	11	30%	34%	44%	45%	52%	42%	62%	43%
O’Malley SS (2008) [32]	67	No Comorbid	NTX + SERT	NTX + PLA	33	34	43.2%	25.29%	96.3%	3.08%	40.6%	26.86%	94.8%	3.09%
Raymond F Anton (2006) [33]	302	No Comorbid	NTX + ACAM	NTX	148	154	22.9%	24.7%	80.5%	25.91%	29.8%	24.7%	80%	26.06%
Raymond F Anton (2006) [33]	300	No Comorbid	NTX + ACAM	ACAM	148	152	22.9%	24.7%	80.5%	25.91%	24.6%	24.78%	75.6%	26.01%
Raymond F Anton (2006) [33]	312	No Comorbid	NTX + ACAM	NTX	157	155	26.8%	24.68%	77.6%	25.94%	23.7%	24.78%	75.9%	26.02%
Raymond F Anton (2006) [33]	308	No Comorbid	NTX + ACAM	ACAM	157	151	26.8%	24.68%	77.6%	25.94%	25.3%	24.7%	78.2%	25.93%
Adamson SJ (2015) [34]	138	Depression	NTX + CIT	NTX + PLA	73	65	25.5%	28.4%	68%	32%	26.1%	26.4%	59.9%	32.1%
Josep Guardia (2011) [35]	62	No Comorbid	NTX + QTP	NTX + PLA	30	32	-	-	96.3%	7.1%	-	-	97.6%	4%
Jacques Besson (1998) [36]	55	No Comorbid	ACAM + DSF	ACAM + PLA	24	31	-	-	55%	42%	-	-	28%	38%
Jacques Besson (1998) [36]	46	No Comorbid	ACAM + DSF	DSF + PLA	24	22	-	-	55%	42%	-	-	31%	30%

**Table 4:** Model results of the meta-regression for the mean difference from combined pharmacotherapy to monotherapy for the percentual abstinence on AUD.

	Estimate	S.E.	Z-Value	*p*-Value	95% CI	
%Male	−8.6545	1.3103	−6.6048	<0.0001	−11.2228	−6.0863
Mean Age	0.2519	0.0972	2.5928	0.0095	0.0615	0.4423
Psychiatric Comorbid	0.2766	0.1091	2.5362	0.0112	0.0628	0.4903
Region (USA vs. Other)	−3.9626	0.5960	−6.6488	<0.0001	−5.1307	−2.794
Associated Naltrexone	2.6221	0.4787	5.4776	<0.0001	1.6839	3.5603
Associated Acamprosate	3.0294	0.4887	6.1988	<0.0001	2.0715	3.9872
Associated Disulfiram	0.8183	0.3698	2.2129	0.0269	0.0935	1.5431
Associated Sertaline	4.2262	0.6568	6.4348	<0.0001	2.9389	5.5135
Associated Topiramate	8.6246	3.0667	2.8123	0.0049	2.6140	14.6352
Associated Citalopram	0.7529	0.5299	1.4208	0.1554	−0.2857	1.7915
Naltrexone Control	1.8796	0.3323	5.6568	<0.0001	1.2284	2.5308
Acamprosate Control	2.2960	0.6085	3.7733	0.0002	1.1034	3.4886
Sertraline Control	3.2263	0.7624	4.2318	<0.0001	1.7320	4.7206

**Table 5:** Results of the Mixed-Effects Model and heterogeneity test for the mean difference from combined pharmacotherapy to monotherapy for the percentual abstinence on AUD.

Mixed-Effects Model (k = 24; tau^2^ Estimator: REML)
tau^2^ (estimated amount of residual heterogeneity): 0.0298 (SE = 0.0134)
tau (square root of estimated tau^2^ value): 0.1726
I^2^ (residual heterogeneity/unaccounted variability): 99.68%
H^2^ (unaccounted variability/sampling variability): 314.52
R^2^ (amount of heterogeneity accounted for): 89.12%
Test for Residual Heterogeneity: QE (df = 10) = 2528.5889, *p*-value < 0.0001
Test of Moderators (coefficients 2:14): QM (df = 13) = 196.1926, *p*-value < 0.0001

## Data Availability

Data included in article/Supplementary Materials/references.

## Supplementary Materials

The following supporting information can be downloaded at: https://www.mdpi.com/article/10.3390/brainsci15060542/s1, Table S1: PRISMA CHECKLIST; Table S2: MOOSE.
